# Invasive Evaluation of the Microvasculature in Acute Myocardial Infarction: Coronary Flow Reserve versus the Index of Microcirculatory Resistance

**DOI:** 10.3390/jcm9010086

**Published:** 2019-12-29

**Authors:** John-Ross D. Clarke, Randol Kennedy, Freddy Duarte Lau, Gilead I. Lancaster, Stuart W. Zarich

**Affiliations:** 1 Department of Internal Medicine, Yale-New Haven Health/Bridgeport Hospital, Bridgeport, CT 06610, USA; freddyduartelau@gmail.com; 2 Department of Internal Medicine, St. Vincent Charity Medical Center, Cleveland, OH 44115, USA; randolkennedy@gmail.com; 3 The Heart and Vascular Institute, Yale-New Haven Health/Bridgeport Hospital, Bridgeport, CT 06610, USA; gilead.lancaster@bpthosp.org (G.I.L.); dr.stuart.zarich@bpthosp.org (S.W.Z.)

**Keywords:** microvascular obstruction, myocardial infarction, fractional flow reserve, coronary flow reserve, the index of microcirculatory resistance

## Abstract

Acute myocardial infarction (AMI) is one of the most common causes of death in both the developed and developing world. It has high associated morbidity despite prompt institution of recommended therapy. The focus over the last few decades in ST-segment elevation AMI has been on timely reperfusion of the epicardial vessel. However, microvascular consequences after reperfusion, such as microvascular obstruction (MVO), are equally reliable predictors of outcome. The attention on the microcirculation has meant that traditional angiographic/anatomic methods are insufficient. We searched PubMed and the Cochrane database for English-language studies published between January 2000 and November 2019 that investigated the use of invasive physiologic tools in AMI. Based on these results, we provide a comprehensive review regarding the role for the invasive evaluation of the microcirculation in AMI, with specific emphasis on coronary flow reserve (CFR) and the index of microcirculatory resistance (IMR).

## 1. Introduction

About every forty (40) seconds, an adult in the United States suffers an acute myocardial infarction (AMI) [1]. Over the last two decades, the standard of care for patients with ST-segment myocardial infarction (STEMI) has been primary percutaneous coronary intervention (P-PCI) within ninety minutes of presentation to an equipped center [2,3]. Unfortunately, opening a culprit epicardial vessel does not always halt ongoing ischemia and portend a more favorable outcome [4]. In contrast, changes which occur at the microvascular level (including reperfusion injury, microvascular obstruction, etc.) following intervention are more comprehensive predictors of outcomes [5].

The landscape of PCI in the stable ischemic heart disease (SIHD) population has shifted from an anatomic/angiographic-guided approach to a physiologic-directed one [6]. Physiologic calculations such as fractional flow reserve (FFR), coronary flow reserve (CFR), instantaneous wave-free ratio (iFR), and the index of microcirculatory resistance (IMR) have allowed interventionalists to better access the microvasculature and make more informed decisions in the catheterization lab [7].

Due to the potential limitations of these microcirculation tools in acute infarct zones, their use in the acute coronary population was met with initial hesitation. However, the ability of these invasive assessment tools to detect microcirculatory dysfunction and obstruction has been validated against the current gold-standard approach—contrast-enhanced cardiac magnetic resonance imaging (CMR) [8]. We aim to provide a comprehensive review regarding the role for the invasive evaluation of the microcirculation in AMI with specific emphasis on CFR and IMR.

## 2. Methods

A systematic literature search was done of PubMed and the Cochrane databases for English-language studies published between January 2000 and November 2019. Studies of interest included randomized controlled trials (RCTs), meta-analyses, systematic reviews and observational studies. Search categories included combinations of “fractional flow reserve”, “coronary flow reserve”, the “index of microcirculatory resistance” and “STEMI” (see Table 1 for full list of search terms). Additionally, the references of selected review articles, meta-analyses and guideline statements were reviewed. Selected articles were agreed upon by all authors.

## 3. The Coronary Microvasculature

### 3.1. Anatomic and Physiologic Principles

**Figure 1:**
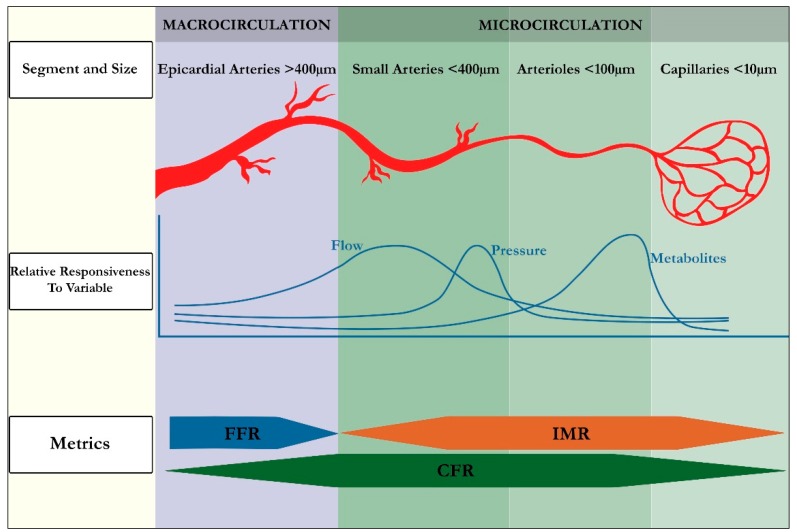
Anatomy and Physiology of Coronary Circulation. FFR = fractional flow reserve; IMR = index of microcirculatory resistance; CFR = coronary flow reserve.

The coronary circulation is an uninterrupted system of vessels of decreasing size, each making unique contributions to meeting the metabolic demands of the downstream vascular bed [9,10]. From the proximal epicardial coronary arteries (>400 µm in diameter) to the small arteries (100–400 µm), arterioles (<100 µm) and capillaries (<10 µm), there exists heterogeneity in resistance control mechanisms (Figure 1) [9,10]. The most proximal vessels, epicardial arteries, have their diameter regulated mainly by forces exerted on their walls by the flow of blood, and make a negligible contribution to flow resistance (in the absence of significant stenosis) [11]. The diameter of small arteries (proximal and distal pre-arterioles) is most responsive to intravascular perfusion and flow changes respectively; while arterioles are sensitive to tissue metabolites. In combination, these pre-arterioles and intramural distal arterioles constitute the major resistance circuit in myocardial blood flow and maintain a constant coronary blood flow over a wide range of coronary perfusion pressures via dynamic changes in diameter. The diameter of capillaries is essentially fixed, and they mainly serve the essential function of nutrient exchange [10].

### 3.2. Pathological Conditions

#### 3.2.1. Coronary Microvascular Disease (CMD)

The term coronary microvascular disease (CMD) comprises a subset of disorders that affect the smallest caliber vessels of the heart and can be the sole contributor to myocardial ischemia; in the absence of visible atheromatous disease [11]. CMD is associated with a series of functional and structural changes in the resistance vessels of the heart that lead to absolute decreases in blood flow and ischemia [12]. The clinical manifestations of CMD are indistinguishable from those of typical angina pectoris, and its equivalents, or other forms of “atypical chest” pain [13]. Clinical evaluation has a very insensitive diagnostic ability, so confirmation is reliant on a variety of non-invasive and invasive tests—either used solely or in combination. Furthermore, it is often a clinical diagnosis of exclusion [11].

#### 3.2.2. Microvascular Obstruction (MVO)

Prompt re-establishment of coronary blood flow after prolonged occlusion does not always lead to shared positive outcomes at the microvascular level [14]. The restoration of perfusion sets off a cascade of events encompassing myocyte swelling, development of endothelial projections that occlude capillaries, platelet-neutrophil recruitment and the deposition of fibrin. This complex pathophysiology has been temporally divided into three phases covering ten relevant factors: (i) pre-perfusion (patient factors, endothelial abnormalities and decreased capillary density), (ii) reperfusion (ischemia/reperfusion, embolization and vasoconstriction), (iii) post-reperfusion (increased endothelial permeability, external compression and inflammation) with dynamics and repair spanning all three phases [15]. Microembolization of atherosclerotic debris following percutaneous interrogation of a coronary lesion further contributes to microvessel occlusion [14]. This narrowing of coronary vasculature is what defines the “no-reflow” phenomenon that will be discussed later. A more comprehensive state-of-the-art review on the remaining aspects of the pathophysiology of MVO is covered in recent work by Rios-Navarro and colleagues [15].

#### 3.2.3. Ischemia/Reperfusion Injury and “No-Reflow”

We have already outlined the contradictory outcomes of primary coronary reperfusion. Although it is known that timely restoration of epicardial flow reduces mortality, we describe downstream consequences which are linked to poor survival [5]. Areas of no-reflow prevent arterial blood and pharmacological agents from entering the ischemic territory [16].

A significant enough perfusion-demand mismatch leads to ischemic cellular injury and death. The ultimate volume of myocardial infarction is directly proportional to the length of time coronary flow is occluded [17]. Irreversible ischemic injury begins to occur after just 15 min of ongoing occlusion [18]. It was first noted in canine models by Reimer and Jennings [19] that transmural ischemic injury resulted in a “wave” of death starting from the subendocardial layer to the subepicardial myocardium. They also noted that if reperfusion was established within 6 h, there was salvable myocardium. An additional finding was that the percentage of tissue necrosis plateaus between 3 and 6 h. This 3 h period may be the most beneficial window for administering reperfusion measures [14].

Ischemia/reperfusion injury results in four categories of myocardial dysregulation [20]. The first type is muscular dysfunction in the absence of irreversible damage (“myocardial stunning”). The second is the microvessel occlusion (MVO) from capillary and myocyte damage. The remaining two are “reperfusion arrhythmias” and “lethal reperfusion injury” [20]. This review is focused solely on the second category-MVO.

The significant contribution that microembolization of coronary debris plays in MVO was alluded to earlier. Microemboli resulting in myocyte necrosis and edema have been noted in as many as 79% of acute myocardial infarction patients [21]. Distal microemboli related to primary PCI has been documented angiographically in 15% to 19% of cases [22,23].

#### 3.2.4. The “Vulnerable” Plaque

A review of coronary tree disease would be incomplete without a brief discussion of the predisposing factors for atherosclerotic plaque thrombosis or rupture (the “vulnerable plaque”). A variety of morphological factors (e.g., plaque cap size, the presence of remodeling or calcification) and functional indicators (e.g., inflammation, antigen expression and enzyme activity) indicate risk [24]. In vivo, these factors can be discerned using non-invasive or invasive techniques. These include optical coherence tomography (OCT), high-resolution intravascular ultrasonography (IVUS) and MRI.

Given the appreciation of the contributions of environmental and host factors to coronary catastrophes (like thrombosis or rupture), the term the “vulnerable patient” was coined as a better representation of risk [24]. Further discussion of these is beyond our intended scope.

### 3.3. Diagnosis of MVO at the Time of Coronary Angiography

#### 3.3.1. Thrombolysis in Myocardial Infarction (TIMI) Flow Grading

TIMI flow grade is a 4-point semi-quantitative system measuring the degree of epicardial reperfusion [25]. It is scored from 0–3. Points are awarded as: TIMI flow grade 0: absent antegrade flow; TIMI flow grade 1: partial contrast penetration beyond an occlusion with incomplete distal filling; TIMI flow grade 2: patent epicardial artery with opacification of the entire distal artery (however, contrast filling or washout is delayed); TIMI flow grade 3: patent epicardial artery with normal flow [14]. Because TIMI flows 0–2 carry no real difference in mortality outcomes, they are treated homogenously angiographically and termed no-reflow [26,27]. However, it is not surprising, given our aforementioned discussion, that MVO is seen in a significant number of patients with TIMI flow grade 3. This created a requirement for other tools to further risk stratify these patients.

#### 3.3.2. TIMI Frame Count (CTFC)

TIMI frame count (CTFC) is a more objective iteration to TIMI flow grading. It measures the number of cine frames required for the contrast material to reach a distal coronary landmark in the culprit artery. A higher CTFC value correlates with slower epicardial flow and increased mortality, regardless of the TIMI flow grade [28]. While angiographic methods such as TIMI and CTFC provide prognostic information, they lack the ability to characterize the coronary microcirculation and thus, global tissue perfusion.

#### 3.3.3. Myocardial Blush Grading or TIMI Myocardial Perfusion (TMP) Grading

A more direct evaluation of the microcirculation consists of measuring the amount of dye that passes into and out of the myocardium, which appears as a “blush” on the screen [29]. The quality of myocardial perfusion can be graded by the quantification of this “blush” during the injection and washout phases [29]. Myocardial perfusion grading adds further prognostic information to patients with TIMI flow grade 3. Confirmation of grade 3 flow on both assessment tools is associated with a <1% mortality risk [30]. This reinforces the central idea that outcomes in acute myocardial infarction not only depend on the level of visible epicardial flow but also on the adequacy of the coronary microvasculature. We will now review the physiologic tools that are available to the interventionalist for the characterization of the coronary microvasculature.

## 4. Invasive Physiologic Assessment of the Microcirculation in the Catheterization Lab

Over the last half century, increasing value has been placed on the physiological consequences of coronary stenotic lesions [31]. Since the breakthrough of selective coronary angiography in the 1960s, it is now well understood that the anatomical description of these lesions is an incomplete assessment of the significance of coronary narrowing. Furthermore, with the growing appreciation for the impact of microvascular dysfunction, the spotlight is being refocused from the macrocirculation to a downstream microscopic level. It is worth reiterating at this point that the following invasive tools are surrogate markers for microvascular damage and that definitive testing is usually required with the non-invasive imaging techniques: contrast echocardiography and the gold-standard, late gadolinium cardiac magnetic resonance imaging (CMR) [15].

Although invasive direct visualization of the microcirculation at the time of coronary angiography is not possible, [10] techniques such as coronary flow reserve (CFR), pressure-derived fractional flow reserve (FFR) and the index of microcirculatory resistance (IMR) have allowed for safe and reliable quantification of coronary blood flow (and the relative contribution of epicardial stenosis to the pressure-flow relationship) in the catheterization lab. Other available tools which are beyond the scope of our discussion are: (i) Doppler-flow-derived indices, e.g., diastolic flow deceleration, systolic flow reversal or coronary flow velocity reserve (CFVR), and (ii) Doppler-flow/pressure-derived indices, e.g., hyperemic microvascular resistance (HMR), zero-flow pressure (Pzf) and wave-intensity analysis (WIA).

The physiological foundation on which all the ensuing invasive techniques are based, requires an appreciation of two key relationships: (1) the relationship between pressure gradient, resistance and flow (Ohm’s law) and (2) myocardial oxygen demand and its consequence on coronary flow.

Ohm’s law states that the resistance in a circuit is directly proportional to the change in pressure and inversely related to the absolute flow.

$$R\;=\;\frac{\Delta P}{Q}$$

where R is the resistance, ΔP is the pressure gradient, and Q is flow. In vivo, epicardial stenosis increases resistance to flow. To maintain myocardial oxygen supply, the microvascular vessels dilate to accommodate these changes. At resting conditions, this has little physiologic consequences, and the microvasculature can tolerate relatively significant coronary narrowing, until a critical point is reached where resting flow is impaired [31]. These significant lesions also result in distal artery pressure loss [32]. However, because the distal circulation is already compensating for reduced supply in the setting of a critical stenosis, when maximal increases in coronary flow are induced (through hyperemia), these distal vascular territories have a relatively smaller increase in the degree of increase in flow, i.e., diminished coronary flow reserve.

### 4.1. Coronary Flow Reserve (CFR)

Coronary flow reserve (CFR) was first described by Gould et al. in 1974. This was the first report of a physiological assessment of coronary stenosis with a hyperemic stimulus. CFR is the ratio of hyperemic to resting absolute flow:

$\mathrm{CFR}\;=\;\frac{\mathrm{Hyperemic}\;\mathrm{Flow}}{\mathrm{Resting}\;\mathrm{Flow}}$. A value of >2.0 is considered normal. CFR accounts for flow through both the epicardial vessels and microvasculature.

**Figure 2:**
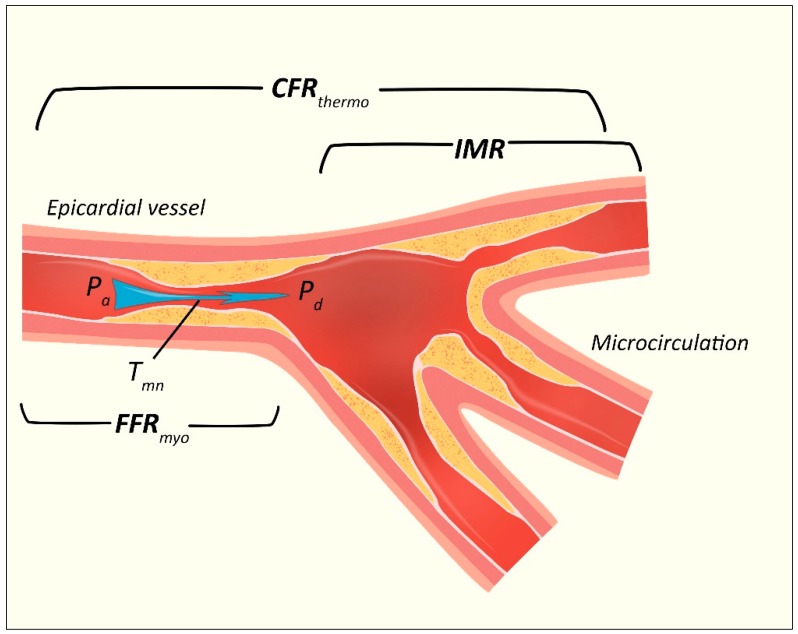
Invasive Assessment of the Coronary Microvasculature. CFR_thermo_ = coronary flow reserve derived from thermodilution; IMR = index of microcirculatory resistance; Pa = mean proximal coronary pressure; Pd = mean distal coronary pressure; T_mn_ = mean transit time; FFR_myo_ = fractional flow reserve (specific to myocardial blood flow).

In its first iteration, CFR was measured using a surgically implanted electromagnetic flow meter with diatrizoate as the hyperemic stimulus (either intravenous or intracoronary adenosine is most often used today). Since that time, CFR measurement tools have gone through a number of invasive and non-invasive technique evolutions including doppler velocity wire sensors (CFR_Doppler_) and, more recently, pressure-monitoring wires using thermodilution (CFR_thermo_) (Figure 2) [33].

CFR measurement via a pressure-monitoring wire using a thermodilution model was not validated until 2001 by de Bruyne and colleagues. Based on the law of thermodilution, flow can be calculated from the mean time it takes a fixed vascular volume (in this case saline) to travel from an injector to a sensor.

$$F\;=\;\frac{V}{T_{\mathrm{mn}}}$$

where F indicates flow; V, vascular volume between the injection site and the sensor; and T_mn_, the mean transit time for the indicator to traverse this distance.

The thermodilution method for assessing CFR assumes a constant coronary vessel diameter [33]. However, in reality, when flow-induced endothelium-mediated vasodilatation occurs this is not the case [34]. An ideal alternative would be simultaneous measurement of the vessel cross-sectional area and mean velocity (V_mean_) to derive the intracoronary blood flow (Q in mL/min) [34]. The Doppler-based wire systems used in physiologic intracoronary measurement directly measure average peak velocity (APV). To calculate V_mean_, a constant coefficient of 0.5 is commonly used, whereby: V_mean_ = 0.5 × APV. Unfortunately, this coefficient does not hold true with pulsatile flow. The average peak velocity (APV) is used to calculate another Doppler-flow-derived index which was mentioned earlier—coronary flow velocity reserve (CFVR).

CFVR is defined as the ratio of APV during hyperemia and at rest:

$$\mathrm{CFVR}\;=\;\frac{{\mathrm{APV}}_{\mathrm{hyperemia}}}{{\mathrm{APV}}_{\mathrm{rest}}}$$

In light of the aforementioned limitations of APV, calculation of CFVR from APV alone has many shortcomings and may lead to misleading results in clinical applications [34]. For this reason (among others), this tool has been largely replaced by CFR_thermo_ in clinical practice in most cardiac catheterization labs.

CFR and CFVR have been the main flow-based parameters used in the investigation of the coronary microcirculation and microvascular obstruction (MVO). Both techniques have been validated in the assessment of: (i) myocardial blood flow (MBF, in mL/min/g) with PET (positron emission tomography) as well as (ii) MVO with cardiac magnetic resonance imaging (CMR) [35]. Due to the similarities in derivation, they are sometimes used synonymously in the literature, which can add to confusion for the reader. For simplicity, we will refer to CFR (CFR_Doppler_) as the Doppler-flow-derived method for most of this review.

#### Limitations of CFR

Despite its excellent performance in assessing myocardial perfusion at multiple levels, CFR has a few inherent limitations. The most significant of these include: (i) it is affected by overall hemodynamics, (ii) it assumes there is negligible contribution from collateral flow and (iii) it is not specific to the microvasculature. To account for the former two limitations, Pijls et al. proposed a more lesion-specific approach based on a pressure-derived estimate of coronary flow [36].

### 4.2. Fractional Flow Reserve (FFR)

Fractional flow reserve is defined as the maximal achievable blood flow through a stenotic lesion divided by the maximal flow that would be achieved if there was no stenosis [37]. It should become clear from the previous equations that for a direct relationship between coronary pressure and flow to be valid, resistance in the circuit (coronary vessel) must be constant and minimal [36]. This is theoretically the case when an epicardial vessel is at maximal hyperemia. At the point of maximal hyperemia there is a direct linear relationship between absolute flow and the inverse of flow velocity ($\frac{1}{T_{\mathrm{mn}}}$) (the significance of this will become more apparent later).

FFR can be calculated from simultaneous measurement of mean arterial, distal coronary, and central venous pressure (P_a_, P_d_, and P_v_, respectively). P_v_ contribution is usually considered negligible (and not measured). FFR is thus, the ratio of the absolute distal coronary to aortic pressures measured during maximal hyperemia. $\mathrm{FFR}\;=\;\frac{P_{d}}{P_{a}}$ (Figure 2), with a theoretical normal value of 1 (P_d_ = P_a_). In clinical practice, a value of <0.75 was originally considered to indicate significant impairment in coronary pressure [38]. This threshold was increased to <0.80 after the outcomes of the FFR versus Angiography for Multivessel Evaluation (FAME 1) trial [39]. The clinical significance of FFR values in this 0.75–0.80 gap therefore generated much attention. The benefit of revascularization in this threshold group unfortunately is inconclusive [40,41,42].

#### FFR vs. CRF: Advantages and Significance of Discordance

The maximal achievable blood flow to the myocardium (FFR_myo_) represents both antegrade and collateral flow—an advantage over CFR. FFR_myo_ depicts the physiologic consequences of a coronary lesion, since the relative supply–demand discrepancy in patients with CAD is what determines symptoms. Unlike CFR, FFR_myo_ measurement is not affected by hemodynamics. One very critical limitation of FFR though is that the model on which it is based assumes a normal microvasculature [36]. FFR_myo_ therefore does not measure microvascular dysfunction beyond the tip of the instrument [37].

Both CFR and FFR are complimentary tools, each gathering valuable information at various points along the vascular tree. Therefore, there is merit in the use of both tools in the same patient. The question is though, what are the implications of FFR–CFR discordant data?

Low CFR in patients with preserved FFR (↑FFR↓CFR) indicates higher microvascular resistance, in spite of similar plaque burden [43]. The addition of CFR adds to the discriminatory ability of measuring plaque burden and microvascular resistance (i.e., low FFR–low CFR (↓FFR↓CFR) denotes higher plaque and resistance when compared to controls (↑FFR↑CFR) [43].

### 4.3. The Index of Microcirculatory Resistance and Instantaneous Wave-Free Ratio (iFR)

The index of microcirculatory resistance (IMR) was reported in 2003 by Fearon et al. [44] in an effort to provide a dedicated assessment of the microcirculation (excluding epicardial vessels, as is the case with CFR). This technique was only feasible because of two technological advancements, (i) the ability to simultaneous measure pressure and flow velocities with the same device [45] and (ii) software that allowed the shaft and pressor sensor of a wire to act as proximal and distal thermistors, respectively.

Using derivations from equations we have previously outlined, resistance in the microcirculation can be derived as follows [46].

$$\mathrm{IMR}\;=\;\frac{P_{d}}{\left(\frac{1}{T_{\mathrm{mn}}}\right)}$$

This can be simplified further to: IMR = P_d_ × T_mn_.

IMR can quantify microvascular function [44]. Normal values are usually reported as ≤25 [47,48]. IMR is not significantly affected by the presence of an epicardial vessel stenosis. Because a standardized coronary pressure wire is used, simultaneous measurement of FFR can discriminate macrocirculatory from microcirculatory dysfunction [44]. An advantage over CFR is its reproducibility [49].

#### Instantaneous Wave-Free Ratio (iFR)

Instantaneous wave-free ratio measures the ratio of P_d_ to P_a_ during an isolated period of diastole (i.e., the “wave-free period”). The wave-free period is the point during diastole when coronary flow is at its peak [50]. iFR is performed independent of adenosine and is therefore termed a “resting index”. One of the attractive properties of iFR is that it can determine similar data to FFR without the adverse effects of hyperemic stimuli. It is unfortunately subject to many of the same limitations as FFR.

## 5. Invasive Physiologic Assessment of the Coronary Circulation in STEMI: What is the Evidence?

**Figure 3:**
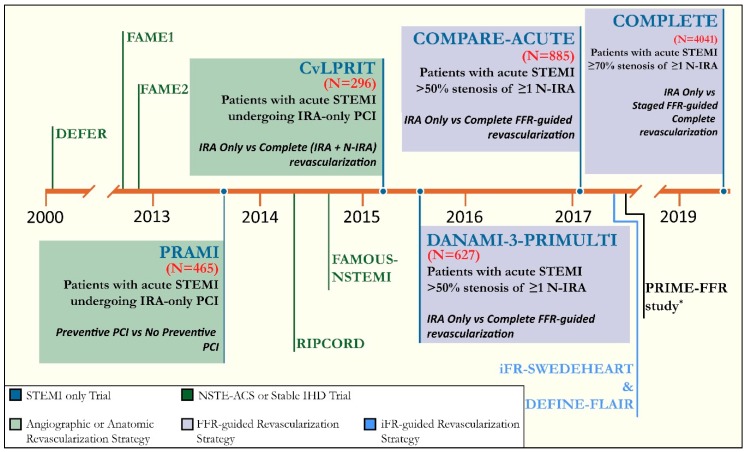
Timeline of Physiologic-Guided Landmark Clinical Trials in Acute Coronary Syndromes using Fractional Flow Reserve. (* prospective registry study). IRA = infarct-related artery; N-IRA = non-infarct-related artery; PCI = percutaneous coronary intervention; NSTE-ACS = non-ST-segment acute coronary syndromes; IHD = ischemic heart disease, FFR = fractional flow reserve, iFR = instantaneous wave-free ratio. FAME = FFR versus Angiography for Multivessel Evaluation.

The role of physiologic-guided PCI in acute coronary syndrome, including STEMI, is an evolving area of research. Most of the large clinical trials over the last two decades have focused on the utility of FFR in acute coronary syndromes (Figure 3). However, the ability of FFR to truly assess the microcirculation is inadequate [11]. Studies using CFR and IMR in reperfused STEMI patients have been limited to small numbers without any head-to-head comparisons (Table 2). In addition, the follow-up periods of these studies have been relatively short. Unfortunately, much of the available trial data showing differences in outcomes such as major adverse cardiovascular event (MACE) rates, recurrent MI or risk of revascularization using physiologic tools is limited to pressure-derived fractional flow reserve (FFR). The physiologic assessment tools CFR and IMR are more dedicated measures of the microcirculation and will be the focus of the remainder of this review.

### 5.1. Invasive Assessment of the Coronary Microcirculation in Reperfused STEMI: Predicting Microvascular Dysfunction and Prognosis

#### 5.1.1. Flow-Derived Index: CFR

Most of the early investigation of CFR utility, was in its ability to assess reperfusion success and predict LV functional recovery post acute MI [52,53]. CFR measurements were compared to echocardiographic assessment early on, followed by SPECT (single photon emission computed tomography) [71] and then CMR [54].

Doppler-derived CFR was a better prognostic tool for determining LV function recovery post AMI, when compared to TIMI flow grade, corrected TIMI frame count (CTFC) and myocardial blush grade [53]. Furthermore, improvements in CFR measures within 24 h of revascularization was predictive of subsequent functional recovery [52]. On the contrary, when Beygui et al. in 2003 used SPECT in combination with contrast ventriculography, relative CFR changes correlated with the extent of the infarct area but not contractility recovery [71]. Prereperfusion anterograde and collateral flows and myocardial viability were better independent predictors of recovery [71].

One of the earliest prospective studies to look at the usefulness of CFR in predicting a hard clinical endpoint after reperfused STEMI was in 2007 by Takahashi and colleagues [54]. After evaluating 133 patients with anterior AMI, they noted that CFR correlated significantly with LV ejection fraction at 4 weeks, and patients with CFR ≤1.3 had higher risk of heart failure and higher incidence of cardiac events [54]. Additionally, low intracoronary Doppler flow velocity measurements in the infarct-related artery after primary-PCI is associated with significantly increased mortality at 10 years [56].

#### 5.1.2. Pressure-Derived Index: IMR

The index of microvascular resistance is not as readily available as CFR but has better reproducibility [11,49]. Fukunaga and colleagues in 2014 reported the clinical outcomes of 88 STEMI patients who had coronary blood flow analyzed by a pressure sensor/thermistor-tipped guide wire within fifteen minutes of successful revascularization [63]. Almost 60% of the patients had evidence of MVO on follow-up MRI at 2 weeks. Although MACE rates were significantly higher in patients with higher IMR values, the presence of MVO and the shape of the IMR thermodilution curve were stronger predictors of outcomes/prognosis [63]. A bimodal curve pattern was an independent predictor of cardiac death at 6 months and was associated with a higher risk of death and heart failure rehospitalization [63].

A larger cohort of 283 patients were followed prospectively for 6 months following reperfusion after STEMI [70]. Carrick et al. sought to determine the utility of using the thermodilution measures of IMR or CFR either alone or in combination to determine MVO and predict outcomes. The primary composite endpoint was all-cause mortality and first heart failure event after hospitalization. An IMR >40 was associated with MVO and was a multivariable associate of myocardial hemorrhage [70]. Independent of infarct size, an IMR >40 was associated with a higher risk of death from all causes, or heart failure. This IMR threshold was associated with LV ejection fraction and end-diastolic volume changes noted on CMR. The CFR cutoff of ≤2.0 did not add prognostic value [70]. Both modalities were able to detect microvascular obstruction and myocardial hemorrhage when measured in the culprit artery but had varying discriminatory ability in patients with differing severity of vascular damage [70].

Cuculi et al. in 2014 reported one of the most insightful prospective looks into the progression of the microvasculature in primary PCI-treated STEMI patients [64]. The authors noted that CFR and IMR at day 1 correlated with the extent of myocardial edema. CFR was significantly lower in patients with MVO at the index procedure and at day 1. However, at 6 months CFR and IMR were comparable between patients with and without MVO. Additionally, IMR at 6 months was comparable to IMR measured in patients with stable coronary artery disease [64]. Although their results were similar to previous studies (Marques et al.) [72], their cohort may not be generalizable since most of the patients had relatively preserved systolic function (mean LVEF 48.0 ± 12%).

### 5.2. Utility of Invasive Assessment Tools in Mitigating Microvascular Obstruction

We have already reviewed the evidence for the ability of CFR and IMR to predict not only functional LV changes but also clinical outcomes after reperfusion. The more important question is whether the information gathered at the time of primary PCI is potentially useful for mitigation strategies. It is worth mentioning that therapeutic interventions to mitigate MVO are most useful early after revascularization (if not during the same procedure) since most of the progression of MVO is related to reperfusion injury [14]. The advantage that invasive assessment tools have over noninvasive assessments (which can be performed up to 5 days later) is that intervention/mitigation strategies are possible at the time of P-PCI. Current intervention strategies include the use of intracoronary streptokinase, sodium nitroprusside, thrombus aspiration and distal protection devices, and a combination of intracoronary abciximab and aspiration thrombectomy [73,74]. A thorough discussion of the therapeutic and pharmacological measures for reperfusion events is beyond the scope of this review [75].

As was previously mentioned, confirmation of TIMI flow grade 3 is not the end of the reperfusion process, since MVO is present in a significant portion of these patients [76]. IMR may be a promising alternative to confirming reperfusion success [62]. We argue that diagnosis of MVO or assessment of risk of progression is prudent for the reasons we previously discussed [77].

### 5.3. The Future Implications of Invasive Microcirculatory Assessment in Myocardial Infarction

The current landscape of microcirculatory assessment tools is not without limitations [78]. The calculations and equipment involved in measurements such as hyperemic microvascular resistance (HMR) and zero-flow pressure (Pzf) are cumbersome and time consuming, making them non-viable options in typical catheterization labs. Also, despite its superior reproducibility, IMR measurements are still subject to interobserver/institutional variability [78]. Bulluck et al. propose that future prospective studies stratify patients’ risk of microcirculatory dysfunction based on IMR values (with a cutoff of >40) after P-PCI, and tailor therapeutic interventions on this basis. Niccoli et al. in their 2016 review, propose that treatment approaches for coronary microvascular dysfunction and obstruction be grouped into different time windows (i.e., before, during and after the catheterization lab) [75]. The authors go further to critically analyze the shortcomings of prior trials of therapeutic interventions, and propose novel approaches for testing in large trials with clinical endpoints [75].

There is a huge discrepancy between the number of basic science studies demonstrating positive therapeutic effects in MVO, and the number of prospective clinical trials in this area with long term follow-up of hard clinical endpoints [79]. There are a few explanations for this, including the difficulties with replicating the complexity of MVO in vivo and the resource utilization required to design such studies [15,79]. However, despite these shortcomings, there is opportunity for study of these therapeutic interventions in, first, animals models and then safety/efficacy studies in humans [15], and the potential role of invasive microcirculatory tools in the management algorithm.

The effects these therapeutic interventions have on MVO will ultimately need to be validated with CMR imaging, but the hope is that CFR and IMR (either alone or in combination) will be viable options to guide decision making on the need for/and timing of instituting these measures in the catheterization lab. The authors share the view that these invasive tools are potentially highly valuable in MVO in STEMI if used appropriately. Further research in large scale multicenter trials will be needed to legitimize these tools in decision making and to measure effects on mortality, LV recovery, and long-term sequelae like heart failure/rehospitalization rates.

## 6. Conclusions

Invasive assessment tools have a valid role in the evaluation of the microvasculature in acute myocardial infarction. Tools such as CFR and IMR have great discriminatory ability in detecting microcirculatory dysfunction after reperfusion and are predictive of clinical outcomes and LV recovery. A major emphasis over the last decade has been placed on investigating the impact of pressure-derived fractional flow reserve specifically. However, more dedicated microcirculatory assessment tools, such as CFR and the index of microcirculatory resistance, exist. More research is needed to determine the impact that these other physiologic measurement tools have in patients with acute myocardial infarction when they are used either individually or in combination.

## Figures and Tables

**Table 1:** Search Strategy Used in the PubMed and Cochrane Trial Databases.

Attempt	Search Terms
1	“fractional flow reserve”
2	“coronary flow reserve”
3	“index of microcirculatory resistance”
4	fractional flow reserve, myocardial infarction[MeSH Terms]
5	fractional flow reserve, STEMI[MeSH Terms]
6	fractional flow reserve, acute coronary syndrome[MeSH Terms]
7	coronary flow reserve, STEMI[MeSH Terms]
8	“cardioprotection”
9	“no-reflow”
10	1 or 2 or 3 or 8

**Table 2:** Studies using Invasive Assessment of the Microcirculation in ST-segment myocardial infarction (STEMI) Immediately after Reperfusion.

Study	Year	(N)	Population	Follow-Up Period	Outcome
Neumann et al. [51]	1997	19	STEMI	2 weeks	CFR shows improvement as early as 1 h after P-PCI in select patients; which continues within 2 weeks.
Lepper et al. [52]	2000	25	STEMI	1 month	Improvement in myocardial perfusion (as indicated by significant ↑ in CFR at 24 h was predictive of LV functional recovery
Bax et al. [53]	2004	73	Anterior STEMI	6 months	Doppler-derived CFR after P-PCI was predictive of long-term global and regional recovery of LV function
Takahashi et al. [54]	2007	118	Anterior STEMI	62 ± 32 months	Patients with a CFR ≤1.3 were more likely to experience acute heart failure or cardiac death
Cuculi et al. [8]	2014	44	STEMI	6 months	Both CFR at P-PCI and the change in CFR over the first day, correlated with myocardial salvage index
Wakatsuki et al. [55]	2000	31	Anterior STEMI	16 ± 2 days	Coronary flow velocity pattern after P-PCI is predictive of global and regional LV recovery
van de Hoef et al. [56]	2013	100	Anterior STEMI	10 years	N-IRA impaired CFVR measured after P-PCI is associated with increased long-term mortality
Fearon et al. [57]	2008	28	STEMI	3 months	IMR after PPCI predicts left ventricular function and recovery at 3 months
Lim et al. [58]	2009	40	Anterior STEMI	6 months	IMR was reliable in predicting myocardial viability and LV wall motion recovery at 6-month follow-up
McGeoch et al. [59]	2010	52	STEMI	3 months	IMR measured acutely predicted LV function and infarct size at 3 months. IMR was higher in patients with MVO on CMR.
Yoo et al. [60]	2012	34	Anterior STEMI	6 months	A higher IMR is associated with worse functional cardiac improvement—measured by regional wall motion score index and LVEF on echocardiography
Payne et al. [61]	2012	108	STEMI	3 months	IMR after P-PCI predicts myocardial salvage, LVEF at 3 months and infarct characteristics (including IS, MVO and myocardial hemorrhage)
Fearon et al. [62]	2013	253	STEMI	2.8 years	IMR at the time of P-PCI is an independent predictor of death alone and death or rehospitalization related to heart failure.
Fukunaga et al. [63]	2014	88	STEMI	6 months	A bimodal pattern on the thermodilution curve, rather than IMR value, was associated with MVO on CMR and worse mid-term clinical outcome.
Cuculi et al. [64]	2014	45	STEMI	6 months	Using univariate analysis, there is a relationship between IMR and infarct size
Baek et al. [65]	2015	113	STEMI	N/A	Age and symptom-onset-to-balloon time were independent determinants of a high IMR.
Park et al. [66]	2016	89	STEMI	3 months	Complimentary IMR and CFR measurements after P-PCI may discriminate myocardial viability and predict long-term risk of MACCE
Faustino et al. [67]	2016	40	STEMI	3 months	IMR appears to be an early marker of cardiac recovery after AMI. Lower IMR was associated with better myocardial GLS acutely
Ahn et al. [68]	2016	40	STEMI	1 week	↑IMR is an independent predictor of MVO. Combined ↑IMR↓CFR_thermo_ are highly predictive of MVO
Bulluck et al. [69] *	2016	246	STEMI	N/A	Weighted mean IMRs of <32 and >41 were discriminatory between the absence or presence of MVO respectively
Carrick et al. [70]	2016	283	STEMI	845 days	An IMR > 40 was associated with predicting changes in LVEF and risk of all-cause mortality and heart failure

(N) = number of patients; CFR = coronary flow reserve; CFVR = coronary flow velocity reserve; IMR = index of microcirculatory resistance; LV = left ventricular; P-PCI = primary percutaneous coronary intervention; N-IRA = non-infarct-related artery; LVEF = left ventricular ejection fraction; IS = infarct size, MVO = microvascular obstruction; MACCE = major adverse cardiovascular and cerebrovascular events; AMI = acute myocardial infarction; GLS = global longitudinal strain; * meta-analysis; N/A = not applicable.
